# Critical Image Identification via Incident-Type Definition Using Smartphone Data during an Emergency: A Case Study of the 2020 Heavy Rainfall Event in Korea

**DOI:** 10.3390/s21103562

**Published:** 2021-05-20

**Authors:** Yoonjo Choi, Namhun Kim, Seunghwan Hong, Junsu Bae, Ilsuk Park, Hong-Gyoo Sohn

**Affiliations:** 1 School of Civil and Environmental Engineering, Yonsei University, Seodaemun-gu, Seoul 03722, Korea; yoonjo15@yonsei.ac.kr; 2 Stryx Inc., Mapo-gu, Seoul 03991, Korea; namhoon.k@stryx.co.kr (N.K.); cto@stryx.co.kr (S.H.); ceo@stryx.co.kr (I.P.); 3 Shinhan Aerial Survey Co., Ltd., Geumcheon-gu, Seoul 08511, Korea; junsu510@shas.co.kr

**Keywords:** critical image identification, incident type definition, smartphone application, emergency situation

## Abstract

In unpredictable disaster scenarios, it is important to recognize the situation promptly and take appropriate response actions. This study proposes a cloud computing-based data collection, processing, and analysis process that employs a crowd-sensing application. Clustering algorithms are used to define the major damage types, and hotspot analysis is applied to effectively filter critical data from crowdsourced data. To verify the utility of the proposed process, it is applied to Icheon-si and Anseong-si, both in Gyeonggi-do, which were affected by heavy rainfall in 2020. The results show that the types of incident at the damaged site were effectively detected, and images reflecting the damage situation could be classified using the application of the geospatial analysis technique. For 5 August 2020, which was close to the date of the event, the images were classified with a precision of 100% at a threshold of 0.4. For 24–25 August 2020, the image classification precision exceeded 95% at a threshold of 0.5, except for the mudslide mudflow in the Yul area. The location distribution of the classified images showed a distribution similar to that of damaged regions in unmanned aerial vehicle images.

## 1. Introduction

Mobile technology and social media are changing the way people communicate in disaster situations, as well as in their daily lives [1]. Social media has emerged as a useful technology that can support disaster management activities, including the rapid detection of socially disruptive events and improvement of situational awareness [2,3]. On social media platforms, online users are classified as a new type of sensor, referred to as human sensors, and the information collected from human sensors can be used to identify crisis information specific to both individual residents and large geographic areas during disasters [4]. Social sensing, which is a method of detecting incidents and analyzing situations from data generated by human sensors, has the advantage of providing near-real-time analysis of an event without burdening human resources engaged in disaster risk management [5,6,7,8]. Social media platforms provide active communication channels in emergencies, and as a result, first responders, decision makers, and the public can use this information to gain insight into a given situation [9].

Posts on social media contain information about urgent situations, but most of them are daily content. A number of studies have been conducted to extract valid information related to emergency situations from the large number of social media posts. Recent studies have detected emergency situations quickly through spatio-temporal analysis of posts [4,7,10] or have detected topics or disaster types from text and image contents to support appropriate rescue and response activities [11,12,13,14,15,16,17]. Disaster responders require both temporal and spatial information about the location, severity, and scale of crisis situations; in particular, location information is essential for understanding the impact of a disaster [18,19]. However, social media users tend not to share their location owing to privacy concerns, and less than 1% of posts on social media have geotag information [10,20,21]. To overcome this, studies have been conducted to obtain the locations of events using textual information mentioned in the content of posts. To extract incident and location information from postings, Fan et al. [10] used the bidirectional encoder representations from transformers (BERT) model for text classification and performed geographical mapping based on the location information extracted from the text. In addition, Liu et al. [22] reported that users at flood sites tended to tweet after moving to a safe place rather than posting directly from the affected site. To overcome this problem, Wang et al. [23] and Liu et al. [22] applied the optimal transport methodology, a domain adaptation technique, to relocate spatially biased georeferenced tweets in historical flood areas. However, problems remain, such as a lack of geotagged data, erroneous inferencing of the crisis location, and false alarms inherent in the posted information [3,24,25].

Recently, crowd-sensing has attracted attention as a means of efficiently supporting disaster management. Crowd-sensing collects information from specific applications or platforms for disaster management purposes and can be used to provide useful information about the impacts of extreme events [26]. In crowd-sensing applications, the desired sensors and technologies can be applied, and precise location information can be collected from specific sensors. Research has been conducted to propose a system architecture that supports disaster management together with information and communication technology (ICT) and cloud computing [24,27,28] and to develop smartphone applications that can support a user-centered design and real-time updates [29,30]. However, verifying and integrating the collected data is one of the issues to be addressed in order to obtain valuable information [26].

When a large amount of data is collected, it is difficult for decision makers to check all data, and to obtain valuable information, it is necessary to verify and integrate the collected data [26]. However, research on data processing and the extraction of valid information from crowdsourced data is insufficient. In the case of crowd-sensing data, a significant amount of data may be collected in an area near an incident, and it is necessary to perform a process of filtering the collected data, similar to that for data collected from social media. However, crowd-sensing data require a technological approach to filtering critical data from the numerous data collected at the emergency site, unlike social media, which classifies emergency-related data from the data collected under normal conditions.

In this study, we propose a streamlined way to effectively, accurately, and promptly recognize the situation when image data are collected from a smartphone during an emergency. As a crowd-sensing application, a smartphone application was designed to collect and share real-time information during a disaster. Videos and images are the most effective data sources for understanding disaster situations [31]. Images can provide specific situational information for estimating risk, need, and damage [7]; thus, the smartphone application was designed to collect video and mobile sensor data easily and to be available across a wide range of age groups. Data collected using the developed smartphone application is processed in two steps based on cloud computing. First, an incident type is detected through a clustering-based approach, and the critical images are classified using spatial analysis techniques for each type of incident. The data collected using the proposed smartphone application were processed and analyzed based on cloud computing. The proposed approach is intended to provide information on the principal locations and images of incidents through geospatial analysis to allow decision makers to effectively recognize the situation in an emergency. To verify the effectiveness of the proposed methodology, it was applied to the Icheon and Anseong regions of Gyeonggi-do, Korea, which suffered damage during heavy rainfall in 2020.

The rest of this manuscript is organized as follows. Section 2 provides an overview of the recently developed related research. Section 3 describes the proposed streamline for collecting crowdsourced data, detecting the event, and processing the collected data to support disaster response activities. In Section 4, application of the proposed methodology is explained, and the experimental results are derived using data collected for the actual disaster-affected area. In Section 5, the research results are discussed, and Section 6 concludes the findings of the paper.

## 2. Related Works

Social sensing technology has been developed and widely applied to disaster scenarios for situational awareness [7]. Research to analyze data collected from the public and detect major events has mainly been conducted on social media data. In this section, we investigated studies related to incident type detection, critical image identification, and spatial analysis to extract disaster information.

### 2.1. Incident-Type Definition

Textual information can be used to track the temporal patterns of keywords and to cluster words into topics [32]. Pohl et al. [33] proposed a TF-IDF-based simple natural language processing step to optimize word selection and compared self-organizing maps and agglomerative clustering for sub-event detection. Pohl et al. [11] proposed a two-phase clustering algorithm using location and time information to automatically identify sub-events occurring in emergency situations. Fan and Mostafavi [21] proposed a graph-based event detection method to identify credible and non-ambiguous situational information related to infrastructure disruptions in social media. The proposed method detects credible situational information by analyzing the similarity between posts and captures spatio-temporal patterns of critical infrastructure events. Fan et al. [10] classified posts into humanitarian categories using the pre-trained BERT model and identified events through graph-based clustering to automatically detect the evolution of disaster across different locations.

### 2.2. Critical Image Identification

In addition to text features, the image figures are also crucial in identifying informative posts [14]. Images can provide information about the severity of damage and the urgency of relief needs of victims in disasters [7,34]. Alam et al. [35] proposed an image-processing system called Image4Act, which was based on the visual geometry group-16 (VGG-16) model, and sought to support relief efforts by collecting, removing noise from, and classifying images posted on social media. Li et al. [36] proposed a method to create a damage detection map based on the VGG-19 model and to measure its severity for images collected from social media. Weber et al. [16] trained the Resnet-18 model to classify images collected from social media into 43 types of incidents using a significant number of class-positive and class-negative datasets, and it could automatically detect incidents from images collected from social media. Feng et al. [37] estimated water level using the body part information of a detected person after classifying the geotagged images related to a flood and then created a flood severity map.

### 2.3. Spatial Analysis

Spatial analysis based on location information can be used to classify meaningful data from the crowdsourced data being collected. Geotagged tweets reporting situational information tend to represent the situation in a specific area, and a large number of geotagged tweets posted in the same region indicates a greater severity of damage and more negative disturbances [7]. This trend is based on the behavioral patterns of citizens when a specific incident occurs, and it can be assumed that the basic patterns of data collected from social media and through crowdsourcing in a disaster situation are similar. Fan et al. [7] applied one of the hotspot analysis techniques, the kernel density approach, to better estimate the size of an event based on this assumption. Hotspot analysis has been used to identify regions with severe damage through the spatial analysis of disasters such as hurricanes, forest fires, and landslides [38,39,40]. In particular, among hotspot analysis techniques, Getis-Ord Gi* can separately identify clustered and random patterns, which enables the classification of valid data [40].

Many technologies have been developed to detect emergencies using text and images included in posts from social media platforms. Data collected from disaster-related crowdsourcing platforms are often similar in types of data collected from social media platforms such as text, image, and video, but there are differences in the distribution and frequency of the collected data. Therefore, we proposed a process to analyze the data collected from the disaster-related crowdsourcing platforms using an algorithm developed for social media.

## 3. Materials and Methods

**Figure 1:**
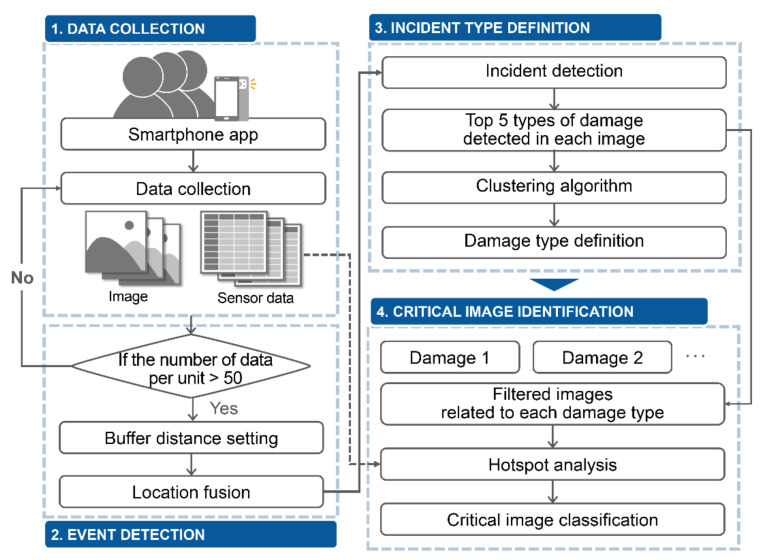
Schematic process for automatic data collection and situation awareness.

In the event of an emergency, the rapid identification of the location and conditions at a site is one of the most important factors determining situational awareness. To support decision-making procedures in emergencies, a holistic process is proposed, from data collection to providing information to decision makers. Figure 1 schematically shows the approach proposed for promptly recognizing a situation using crowdsourced data in an emergency. The method that is proposed for extracting critical information from crowdsourced data is divided into four major steps.

The first step is real-time data collection using a smartphone application. To collect crowd-sensing data efficiently, a smartphone application was developed. Information can be collected in real time from the incident site using smartphones. The second step is event identification. If more than a certain amount of data is aggregated, it can be considered that an event has occurred. Therefore, an area for performing the analysis is set based on the collected location information. The third step is the delineation of the main incident type. Different types of major damage were detected using the aggregated data over the analysis area. The final step is the identification of the principal image. For each type of major damage, images that reflect the situation well are classified, and the location of major damage is identified using the location information of the classified images. Based on the proposed methodology, prompt situation awareness can be achieved, and decision making can be supported. Each step is explained in the following sections and subsections.

### 3.1. Data Collection Using Smartphone Application

This section introduces a smartphone application developed to collect crowdsourcing data from the public and describes a streamlined way to collect data based on a cloud server and provide it to decision makers.

#### 3.1.1. Smartphone Application Development

Owing to the rapid increase in smartphone use and the development of related applications, smartphone applications are widely used in the private sector as well as in the public domain [41]. Recently, mobile crowd-sensing has attracted increasing attention owing to its suitability for a new type of context-aware application and a wide range of services [42]. Mobile crowd-sensing based on mobile devices enables the collection of local spatial information and knowledge, and its dissemination to other users and communities [42,43,44]. Accordingly, using smartphone applications, on-site information can be crowdsourced through local people involved in evolving situations.

A smartphone application was developed for real-time information sharing of emergency situations. Because the data collected along with the location information are shared through the cloud server, the smartphone application can run only when the network and global positioning system (GPS) are connected. When the application is run, the camera screen is the main interface, and the sensor information collected through the application can be checked. Smartphone sensors such as GPS receivers, accelerometers, gyroscopes, and magnetometers are the main sources of data. Using the data collected from the smartphone, the location and direction from which the images were taken can be recovered, and the locations of the images are then displayed on a web-based map.

#### 3.1.2. Processing and Utilization of Collected Data

**Figure 2:**
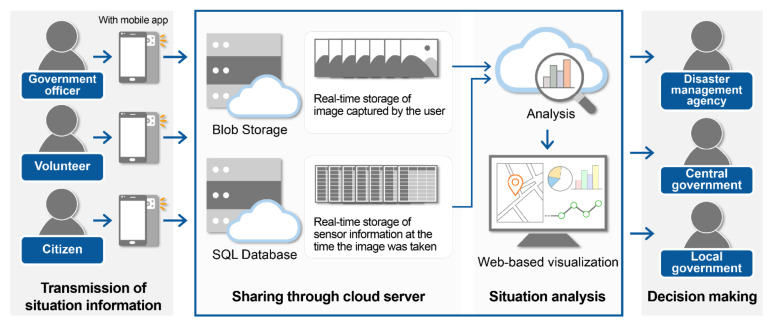
Cloud computing-based automatic situation awareness process using smartphone application.

The proposed streamline using a smartphone application is shown in Figure 2. It basically describes data flows, including data collection, data sharing, data analysis, web-based visualization, and information delivery to decision makers. Data can be collected by public officials who check and investigate the site when an incident occurs, volunteers who report emergency situations and support rescue activities, and citizens in the vicinity of the emergency situation. When a user notices a serious situation and takes images of a dangerous spot using the developed app, those images along with the smartphone sensor data are transmitted to the cloud server automatically. As the app is developed for easy operation, it can be readily used by the private sector, such as local residents, as well as public officials who are responsible for reporting and recording the situation at the disaster site.

The patterns of data collected in normal situations and emergencies may be quite different. While there are little or no data collected in normal situations, when an emergency situation occurs the amount of data can rapidly increase in a short time. Depending on the scale of the disaster and the number of citizens near the scene, the amount of data collected may vary significantly, making the availability of a flexible data storage method essential. Cloud computing technology can be a good solution owing to its flexible and distributed resource allocation through the Internet environment [45]. In particular, when developing an emergency management system, fast data transfer and effective resource utilization are key factors for bridging the gap between technology and first responders [28].

Cloud computing is a large-scale distributed computing architecture that provides storage and computing services to public institutions and individuals over the Internet [46]. The public cloud is the most common type of cloud computing deployment and includes services such as Amazon’s Amazon Web Services (AWS) and Microsoft’s Azure. Through public cloud services, users can allocate and use as many cloud resources as they require, with unlimited scalability and low cost [28].

Microsoft’s Azure cloud service was used in this study. The image data collected from the smartphone application are stored in Azure Blob Storage, which is an object storage solution for the cloud and is optimized to store massive amounts of unstructured data [47]. The collected sensor data are stored in the Azure Structured Query Language (SQL) Database, which provides the necessary SQL databases and fills them with tables and data [47]. Data collected by users can be stored on cloud servers and analyzed based on cloud computing using data collected at specific spatial units. Algorithms to analyze the data within the cloud server are described in detail in Section 3.2, Section 3.3 and Section 3.4. The results of the analysis are visualized on a web-based map and shared with disaster management agencies, central governments, and local governments to support prompt and appropriate decision making.

### 3.2. Event Identification

To efficiently perform situational awareness based on crowdsourced data, it is critical to set a criterion for recognition as an event by using the pattern of data collected in an emergency situation. To achieve a balance between information redundancy and mapping accuracy when analyzing social media data, previous studies used methods such as merging collection data based on the distance between location information extracted from posts and performing analysis by generating polygonal grids based on spatial regions [4,10]. In the case of social media, where activities are conducted periodically, event sensing through the comparative analysis with usual sensing patterns may be a solution. However, in this study, a disaster-related smartphone application was developed, and an analysis was performed based on the data collected. In cities, a large amount of data can be collected because many people are concentrated in localized areas, while in the suburbs, even if a disaster occurs, the amount of data collected may be small because the number of residents in the affected area is small. Therefore, the density of data collected when an event occurs is related to the number of people living in the area, which can have significant implications in event detection.

The penetration rate of smartphones in Korea was approximately 95% in 2018, the highest level worldwide, and the penetration rate of smartphones for users over the age of 50 has reached 91% [48]. With the widespread use of smartphones, information collected by the private sector is extremely useful in various fields, and when it is difficult for reporters to quickly move to a scene, such as after traffic accidents or disasters, there have been cases when they used videos or images directly recorded with the smartphones of citizens [49]. In the United States, the news network CNN launched iReport in 2006 as a portal through which citizens can voluntarily post and receive information on emergency events, and the number of videos and photos posted exceeded 200,000 in two years [50]. YTN, which is a Korean news broadcasting channel, started operating MJ (Mobile Journalist) in 2015, and in 2016, the number of reported items using mobile and online portals reached 30,000 [49]. As such, the following methodology was proposed under the assumption that most citizens own smartphones and that reporting activities are frequent.

In Korea, the output area (OA) is used as the minimum unit boundary for statistical information by Statistics Korea. Population is applied as a basic factor when setting the OA, and the OA is optimally set to 500 people (minimum 300 people, maximum 1000 people) [51]. Assuming that approximately 10 people in an OA each take about five images, more than 50 data can be collected, and more than 50 data collections per OA are proposed as an analysis performance criterion to recognize that an incident has occurred. Depending on the magnitude and location of the event, crowdsourced data can be collected in a single OA or across multiple OAs. Therefore, it is necessary to establish criteria for performing an analysis by merging data corresponding to the same event. The OA has different spatial sizes in cities and suburbs, and it can be set to be very small in areas with high population densities, such as apartment complexes. The extent to which the collected data can represent the corresponding OA can be expressed in the form of a buffer. When setting the buffer of the collected data, it can be set in proportion to the area of the OA containing the data. The size of the buffer zone is set based on the location information of the collected data, and the data where the generated buffers overlap are merged. Based on this criterion, it can be determined whether an emergency situation has occurred in a specific area and whether there is a need to continuously monitor the situation after event detection.

### 3.3. Definition of Major Incident Type

**Figure 3:**
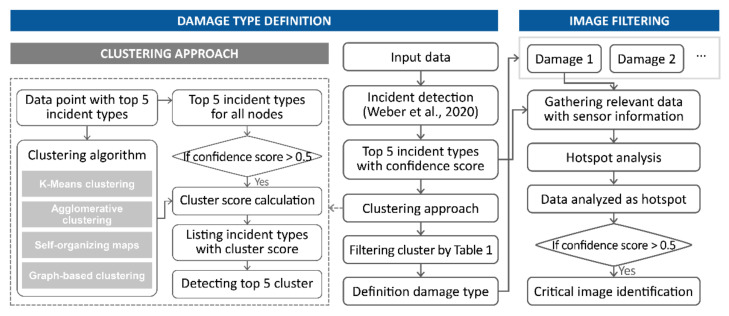
Process of data processing for detecting damage types and image filtering.

In social media, as many users share images and textual information about a given incident site in real time over various regions, it is possible to acquire an overwhelming amount of data compared to other means. Thus, existing studies focus on developing algorithms for classifying disaster-related data and filtering unnecessary data. Recently, deep learning-based computer vision methodologies have been expanding into various fields with excellent performance. However, most machine-learning technologies are limited in their ability to precisely detect and filter related images representing real-time situations and damage [7]. In addition, disaster sites can be described in different ways depending on the magnitude of the damage, regional characteristics, and weather conditions; moreover, in the case of multiple types of damage, it can be difficult to clearly classify a site using simple classes. To address this uncertainty, an incident type is defined using images collected for a set area, and a process of filtering data using spatial analysis according to each type of incident is proposed, as shown in Figure 3. Contents on data filtering using spatial analysis are described in Section 3.4.

#### 3.3.1. Incident Detection Algorithm

As discussed above, several algorithms have been developed for extracting disaster information from images, but most of the research has been conducted to classify disaster-related images from images uploaded daily to a social network. Related studies have divided major incidents into five or six categories (e.g., earthquakes, fires, and floods) with limited types [52,53,54]. However, depending on the risk factors and local characteristics, damage caused by major incidents can be interpreted from various viewpoints. In addition, when a disaster occurs, various types of incidents can occur in the form of complex situations. Therefore, to accurately understand the damage situation, a category-based analysis that considers sufficient incident types should be performed.

Weber et al. [16] reported that existing incident datasets are not sufficient to perform incident detection in terms of both the number of images and categories. To overcome this, a larger, more complex, and diverse incident dataset was constructed by labeling 446,684 positive images and 697,464 negative images for 43 incident categories [16]. To perform training using this dataset, a convolutional neural network (CNN) with two task-specific output layers was used, with Resnet-18 [55] used as the backbone. To solve the classification problem for negative images by approaching the detection method, a class-negative loss was introduced. Class-negative loss is a modification of the existing binary cross-entropy loss, and a weight vector is added to toggle the loss using a partial label consisting of the class-positive and class-negative labels [16]. As such, Weber et al. [16] proposed a powerful model that can detect accidents in the wild using a large, complete, and diverse dataset that classifies the output into 43 incidents and 49 places, and includes the confidence score for each classified result.

In this study, the pre-trained model built by Weber et al. [16] was used, and based on this, the major types of incident were defined and principal images were selected. Weber et al. [16] analyzed the incident classification accuracy using a test set with positive labels, and found that the top-1 accuracy was 77.3% and the top-5 accuracy was 95.9%. Therefore, the incident type (with confidence score) was extracted up to the 5th rank for each image, and it was used as an element to proceed with clustering.

#### 3.3.2. Clustering Approach

An emergency situation may not necessarily be related to one large accident, and many side events in different regions at different times may occur in a large-scale crisis [11]. Pohl et al. [11] detected sub-events using a clustering method that does not require labeling because people use different terms to describe crises that have their own characteristics such as area of occurrence, scalability, and impact. The data configured for clustering is composed of the top-5 incident types and confidence scores for each incident. In this study, the top-5 incident types and confidence scores were extracted from each image, and the four clustering algorithms were used to detect the types of incidents in the analysis area.

K-means clustering algorithm has been used to detect topics in social media data owing to its efficiency and easy implementation [13,56,57]. The K-means clustering is an unsupervised clustering algorithm that finds groups in data. Given the observations, n observations are partitioned into $k\left(\leq n\right)$ clusters by minimizing the within-cluster sum of squares, which is defined as the sum of the distances of each point in the cluster for k centers.

Pohl et al. [33] used self-organizing map and agglomerative clustering for sub-event detection from social media data and applied it to datasets with four different characteristics, confirming that both algorithms identified important sub-events. Agglomerative clustering is hierarchical clustering, and the most similar clusters are merged based on the distance measure at each step [58]. In the initial stage, each input is set as an individual cluster, and the process of merging similar clusters ends when the specified number of clusters is obtained and returns the cluster [58]. A self-organizing map is an artificial neural network based on an unsupervised learning method, and it does not require prior knowledge of data distribution. The self-organizing map reduces the dimensionality of multi-dimension feature vectors and performs clustering at the same time [11]. Visualizing complex non-linear multivariate data in a two-dimensional space brings the advantage that the pattern of the data can be clearly expressed.

In K-means clustering and agglomerative clustering, which require the designation of the number of clusters in advance, the number of clusters was designated as five. After performing the clustering, there are still various incident types mixed in each cluster. Therefore, it is necessary to define the types of incidents reflected by each cluster. When each of the top-5 incident types extracted from each image was referred to as content, the content with a confidence score lower than 0.5 for all contents was removed. By calculating the number of incident types remaining for each cluster, the incident type with the highest frequency is defined as the incident type corresponding to the cluster. At this time, if there is a cluster designated as the same incident type, it is considered as one cluster.

Tweets, including credible situation information, tend to be retweeted repeatedly by other users in a short period of time [7,10], and the reliability of contextual information can be determined based on the similarity of content between tweets posted by different users [59]. Fan and Mostafavi [21] proposed a graph-based event detection method to identify reliable and unambiguous situational information about a specific incident from social media, and a graph-based clustering methodology was used to identify critical tweets [7,10]. Graph-based clustering examines the distance between each data and builds semantic graphs based on their similarities. Distance is measured using cosine similarity. After calculating the sum of the weights connected by each node in the configured graph, giant components are detected and identified as credible and critical posts. As in the case of applying the aforementioned clustering method, when a graph is constructed using the data used in this study, each node includes the top-5 incident types. Therefore, as before, all contents with a confidence score lower than 0.5 were removed. For remaining content, the sum of the weights calculated at the node containing each content was considered as the score of each content. When the sum of the content scores according to incident type was calculated, the highest incident type was detected up to the top 5.

#### 3.3.3. Categories of Incident Types

The model proposed by Weber et al. [16] includes 43 incident categories, including non-emergency categories such as under construction, dirty contaminated, and traffic jam. Because the purpose of this study is to recognize emergencies promptly, the categories proposed by Weber et al. [16] were re-categorized to detect major incident types corresponding to emergencies.

The ultimate goal of classifying images according to damage type is to enable the authorities to direct appropriate resources for each situation [52], and Mouzannar et al. [52] and Amin et al. [53] divided damage into five categories based on this goal in order to classify the data collected from social media: (1) infrastructural damage, (2) damage to natural landscape, (3) fire damage, (4) flood damage, and (5) human damage. In the case of human damage, it provides important information that requires prompt response, but we did not consider it because the purpose of our study was to classify principal images based on geospatial analyses, and to transmit information about the location of heavily damaged areas. In order to support decision makers in performing appropriate response activities, the 43 incident categories of Weber et al. [16] were re-categorized based on the categories proposed by Mouzannar et al. [52], and the re-categorization result is listed in Table 1. Among the incident types extracted through analysis, only the incident type corresponding to “main” was selected.

### 3.4. Image Filtering Based on Geospatial Analysis

When the amount of data is not large, information pertaining to the emergency situation can be provided based on the confidence score for the detected incident type. However, when the amount of collected data increases to several hundred data or more, the decision maker cannot check all images, and the analysis using the incident detection algorithm can lead to different results depending on the local conditions. Therefore, key situational information that can support efficient decision-making should be filtered and provided to decision makers. The key objective of this procedure is to identify the major affected areas and to classify the principal images to support efficient decision making.

The Getis-Ord Gi* [60] methodology was used to identify the locations of hot spots. Gi* has the advantage of being able to test statistical significance by calculating the z-score, and it is very useful because it serves as an indicator of local spatial autocorrelation [61]. This method evaluates the degree to which features with similarly high or low values exist around each feature [62]. In the heat map obtained as a result of the analysis, statistically significant spatial clusters can be identified for both hot spots (with high cell values) and cold spots (with low cell values). Getis-Ord Gi* can be calculated using Equation (1).

(1) $$G_{i}^{*}=\frac{\sum_{j=1}^{n}w_{ij}x_{i}-\bar{X}\sum_{j=1}^{n}w_{ij}}{S\sqrt{\frac{n\sum_{j=1}^{n}w_{ij}^{2}-{\left(\sum_{j=1}^{n}w_{ij}\right)}^{2}}{n-1}}}$$

where S is the standard deviation, $w_{ij}$ is the value of the spatial weight matrix, n is the total number of samples, $x_{i}$ is the attribute value of object j, and $\bar{X}$ is the average value of the space unit. i and j refer to the units of space of an individual entity. For each incident type analyzed above, hot spot analysis was performed, and the attribute value of each point was assigned a confidence score for the incident type extracted from the image.

## 4. Case Study of Heavy Rainfall in 2020 in the Korean Peninsula

### 4.1. Study Area and Dataset

In Korea, the rainy season in the summer of 2020 lasted for a total of 54 days, starting on 24 June and ending on 16 August in the central region, ranking as the longest rainy season since 1973, when meteorological observations were expanded nationwide [63]. An average of 858 mm of precipitation occurred across Korea, excluding Jeju Island, which accounted for about 66% of the annual average of 1299.7 mm [63]. It caused severe flood damage across the country, and in August, 38 cities and 36 towns were declared special disaster areas as a result of heavy rainfall [63]. To verify the proposed processes, the study areas were chosen considering the accessibility of data acquisition as the key factor. The study areas were Sangyang-ri, Yul-myeon, Icheon-si (Yul), Hwabong-ri, Iljuk-myeon, Anseong-si (Iljuk), and Jangwon-ri, Juksan-myeon, Anseong-si (Juksan) located in Gyeonggi-do, among the areas affected by the heavy rainfall on 2 August 2020.

Some sections of the bank of the Sanyang Reservoir in Yul-myeon collapsed and the Sanyangcheon Stream overflowed; as a result, the entire Sanyang 1-ri village located under the reservoir was submerged, and more than 10 households flooded [64]. In Iljuk-myeon, the soil that was shifted by a landslide hit a poultry farm and a house, leading to a casualty [65]. In Jangwon village in Juksan-myeon, the foundation of the mountain behind the village was weakened and the soil was swept away, resulting in seven households being damaged or buried [66].

Data were collected using a smartphone application at the affected area on 5 August and 24–25 August after the accident. On 5 August rainfall continued, and the debris was being cleaned up, but the damage remained. During 24–25 August, the rainy season was over, the disaster waste had been cleaned up to some extent, and restoration was in progress. The mobile devices used for data collection were Galaxy S7 Edge, Galaxy S8 Plus, and Galaxy S9 manufactured by Samsung.

**Figure 4:**
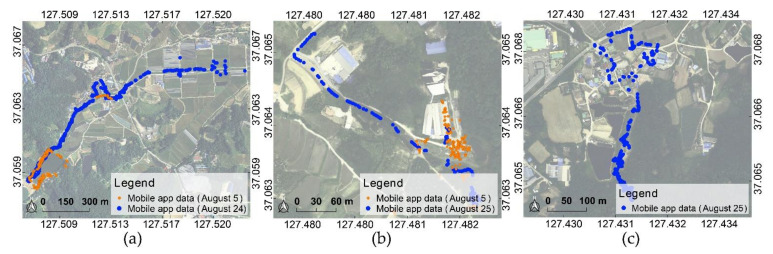
Smartphone application data acquired from study area: (**a**) Yul, (**b**) Iljuk, and (**c**) Juksan. The coordinates refer to WGS84 (EPSG: 4326).

The smartphone application data used for the analysis included 1473 images from Yul, 638 images from Juksan, and 468 images from Iljuk. The data collected on 5 August provided extensive data on the affected areas, and the data collected during 24–25 August were largely from major damaged and surrounding areas. The data collected per region are shown in Table 2, and the distribution of the collected data is shown in Figure 4. The background maps in Figure 4 are aerial orthoimages obtained from the National Geographic Information Institute. In addition, during 24–25 August, FireFly6PRO (BirdsEyeView Aerobotics, NH, USA) equipped with SONY ILCE-6000 (Sony, Tokyo, Japan) was used to acquire unmanned aerial vehicle (UAV) images of the study area. To compare the results of the analysis with the actual location of the damaged area, a UAV-based orthoimage was generated using Agisoft Metashape software, and was used as a background map to verify the analysis results.

### 4.2. Result of Incident-Type Definition

Analyses were performed according to the data collection date and region, and an incident detection algorithm [16] was used to extract incident types and confidence scores for up to five ranks for each image. When considering the incident types extracted for the top-1 incident type, for the data collected on 5 August and 24 in Yul, 20 and 25 incident types were extracted, respectively, and for the data collected on 5 August and 25 in Iljuk, 14 and 21 incident types were extracted, respectively. For the data collected on 25 August in Juksan, 17 incident types were detected. Out of 43 incident types, 31 incident types were detected for the five cases.

**Figure 5:**
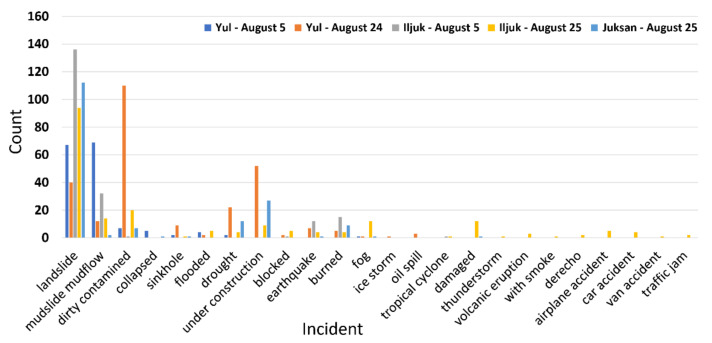
Number of images with confidence score over 0.5 for top-1 incident.

Weber et al. [16] filtered images with confidence scores of 0.5 and 0.9 for Flickr and Twitter images, respectively, and the same criteria were applied to the images collected from our study area. Figure 5 shows the number of images with a confidence score of 0.5 or higher for the top-1 incident type of the collected images. Although many images are included for the incident types related to the real damage situation, there is a limit to specifying the damage types. For the top-1 incident type with a confidence score of over 0.9, mudslide mudflow (5 August, Yul), burned (5 August, Iljuk; 25 August, Juksan), and under construction (24 August, Yul; 25 August, Juksan) were detected, but these results were insufficient to reflect the entire disaster scenario. The information extracted from each image does not compensate for the uncertainty of the machine-learning technique, and an additional filtering procedure is necessary to extract accurate information.

The top-5 incident types detected from images were set as keywords for data, and a clustering approach was applied. Four clustering methods were used: K-means clustering, agglomerative clustering, self-organizing map, and graph-based clustering. Table 3 shows the incident types extracted according to each clustering approach.

In most cases, landslides were identified as the most important cluster. In the images collected on 5 August, mudslide mudflow was detected as a major cluster as the soil that was swept away owing to continuous rainfall was not cleaned up. However, in the case of the images from 24–25 August, a large portion of the dirt that was pushed down was in the process of being cleaned up, and dirty contaminated and drought were detected as some dirt remained in a dry state. In the case of Iljuk on 5 August, earthquake was detected as an accident in which a poultry farm and house collapsed. In the case of Yul and Juksan, which were damaged villages, under construction was detected as a type of incident as restoration activities were being carried out during 24–25 August.

In order to extract incident types related to emergency situations, the results of extracting only the incident types corresponding to “main” in Table 1 are indicated in blue in Table 3. It can be found that not only incidents such as dirty contaminated, under construction, drought not related to emergencies, but also incidents of burned and sinkhole not related to the study area are excluded.

Comparing according to the clustering method, flooded was not detected when K-means and agglomerative clustering algorithms were applied to Yul data on 5 August. For Yul on 24 August, Mudslide mudflow was not detected only when K-means clustering was applied. Incident types that vary depending on the clustering method can be said to be a type related to the field situation, although it is ambiguous to be considered as the main type. Except for these two cases, the same incident types were detected, and analysis was performed on all finally detected types.

### 4.3. Result of Principal Image Classification

**Figure 6:**
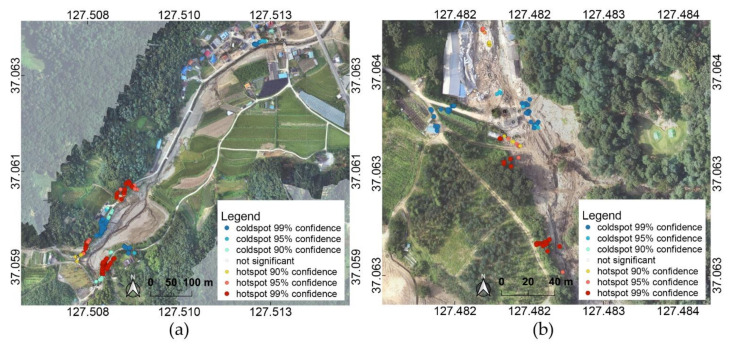
Hotspot analysis on landslide incident using 5 August data: (**a**) Yul and (**b**) Iljuk. The coordinates refer to WGS84 (EPSG: 4326).

All of the images included in the incidents in the 1st to 5th priority, in which each defined incident was detected from the images, were targeted, and the main event location and principal images were identified through spatial analysis for incident sets for each case. In the hot spot analysis, the location information was used as the sensor data collected from the smartphone application, and the attribute value used the confidence score for the incident to be analyzed. The hotspot analysis results for the landslide incident for Yul and Iljuk on 5 August are shown in Figure 6. Analysis results are analyzed as “hotspot,” which has concentrated high values, and “coldspot,” which has concentrated low values; images which were included in a clustering of hotspots with confidence intervals of 90%, 95%, and 99% were targeted.

Given that there are images representing various degrees of severity, providing images reflecting the serious situation to support decision-making can contribute to appropriate response activities by authorities. The evaluation was performed based on the severity of the event in which the final classified images were included. Ground truth data were created by manually classifying the level of damage in the collected images into five categories: very heavy, heavy, moderate, slight, and none. A threshold value was applied to the confidence score of the images extracted through hotspot analysis, and the degree of severity of the classified images was analyzed according to the change in the threshold value. The severity of the images classified according to the change in the threshold value was compared for the results with and without hotspot analysis; these are shown in Table 4 and Table 5, respectively.

In both cases, the number of classified images decreased as the threshold increased, and a number of images with categories corresponding to moderate, slight, and none were excluded. With respect to the number of classified images, a smaller number of images were classified when hotspot analysis was performed, and images were effectively filtered according to the severity of the classified images. However, in the case of the earthquake in Iljuk on 5 August, based on hotspot analysis, the spatial distribution of images corresponding to the incident was insignificant, and related images were not classified. In the case of Iljuk, both the collapse of the structure and damage to the sediment flow resulted from the landslide, and these damages were included in the collected images. When multiple damage types are included in one image, it is difficult to predict which damage type the incident detection algorithm will interpret as having more weight. For the result of the earthquake incident without hotspot analysis, the number of images detected with a confidence score above 0.5 is very small, which indicates that the images were analyzed with an emphasis on landslide damage in the model.

**Figure 7:**
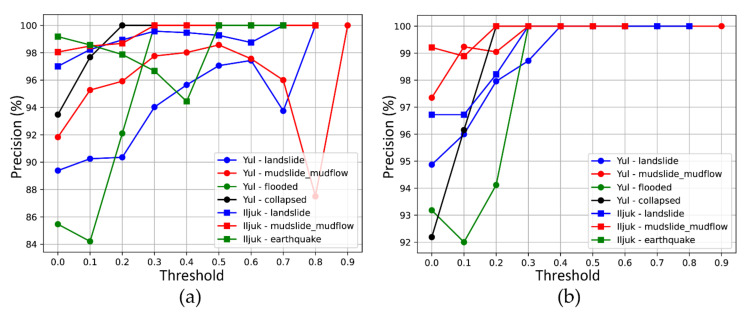
Evaluation of principal image classification using 5 August dataset: (**a**) result without hotspot analysis and (**b**) result with hotspot analysis.

**Figure 8:**
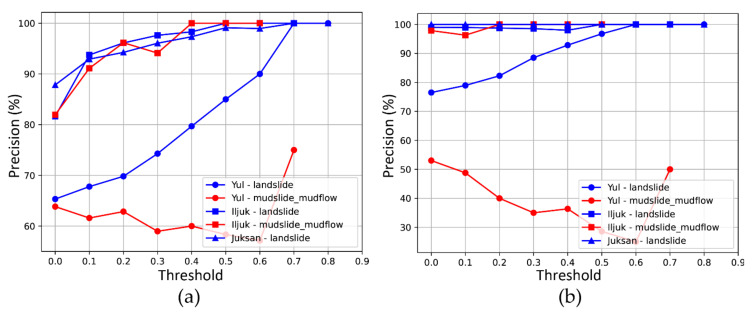
Evaluation of principal image classification using 24–25 August dataset: (**a**) result without hotspot analysis and (**b**) result with hotspot analysis.

To evaluate the precision of the classified images, those classified as very heavy and heavy were regarded as true, and the ratio of the images corresponding to true among the classified images was calculated. The results of analyzing the image classification precision according to the change in the threshold are shown in Figure 7 and Figure 8. From the 5 August dataset, without hotspot analysis, the true proportion tends to be irregular, while with hotspot analysis, except for the aforementioned earthquake in Iljuk, all images with a confidence score above 0.5 were classified as having incurred serious damage.

For the 24–25 August dataset, the precision tends to increase in a relatively stable manner as the threshold changes, except for the mudslide mudflow in the Yul region. Restoration was in progress at the time the data were collected, and a high confidence score was detected for some disorganized dirt piles. Except for this case, the confidence score-based image classification appears to be significant for analysis of the 5 August dataset. Using hotspot analysis, the classification precision was improved by up to 11.77% in the landslide case in Yul, but decreased by 29.76% in the mudslide mudflow case in Yul.

### 4.4. Result of Identification of Major Damaged Location

To determine whether the classified images are located in the area where the major damage occurred, the UAV orthoimages generated from the images taken on 24–25 August were used as a reference map and compared. After analyzing the precision of the classified images according to the threshold, it was confirmed that a significant number of non-damaged images were excluded when the threshold was 0.5. Therefore, the spatial distribution was analyzed using images filtered with a confidence score above 0.5.

**Figure 9:**
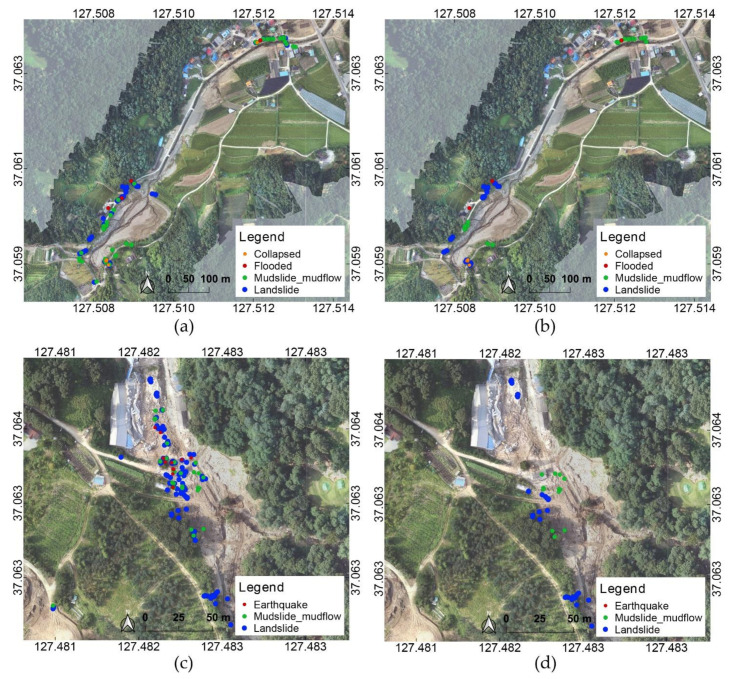
Distribution of results based on analysis of 5 August dataset: (**a**) results without hotspot analysis in Yul, (**b**) results with hotspot analysis in Yul, (**c**) results without hotspot analysis in Iljuk, and (**d**) results with hotspot analysis in Iljuk. The coordinates refer to WGS84 (EPSG: 4326).

The analysis results of the data collected on 5 August are shown in Figure 9. The distribution of results according to the incidents shows a relatively clearly separated distribution trend in the case with hotspot analysis. However, for the case without hotspot analysis, various incidents are mixed spatially, making it difficult to determine the region mainly affected by each incident. The results of the Yul region were analyzed as landslides in the area where the upper part and the bank collapsed around the Sanyang Reservoir, and it was observed to be similar to the damage type of the landslide due to the collapse of soil. In the middle part, the incident type was determined to be mudslide mudflow, and soil covered the village as water escaped from the Sanyang Reservoir.

From the result of the Iljuk, landslides were analyzed in the upper, middle, and lower areas where landslide damage occurred, and mudslide mudflow was analyzed in the middle area. The entire range of the actual landslide occurrence is included in cases both with and without hotspot analysis, but when hotspot analysis was performed, the number of images that were classified was much lower than half. When hotspot analysis was not performed, images corresponding to the earthquake were included; however, in the hotspot analysis, a significant hotspot could not be analyzed. From the result without hotspot analysis, it can be seen that landslide and mudslide mudflow are mixed in the area where the earthquake was detected. The number of earthquake-detected data was 244, of which approximately 70% or more had confidence scores less than 0.1. Owing to the concurrent occurrence of landslide and mudslide mudflow, the confidence score for earthquakes was relatively low; accordingly, it was considered that a significant hotspot analysis result was not obtained.

**Figure 10:**
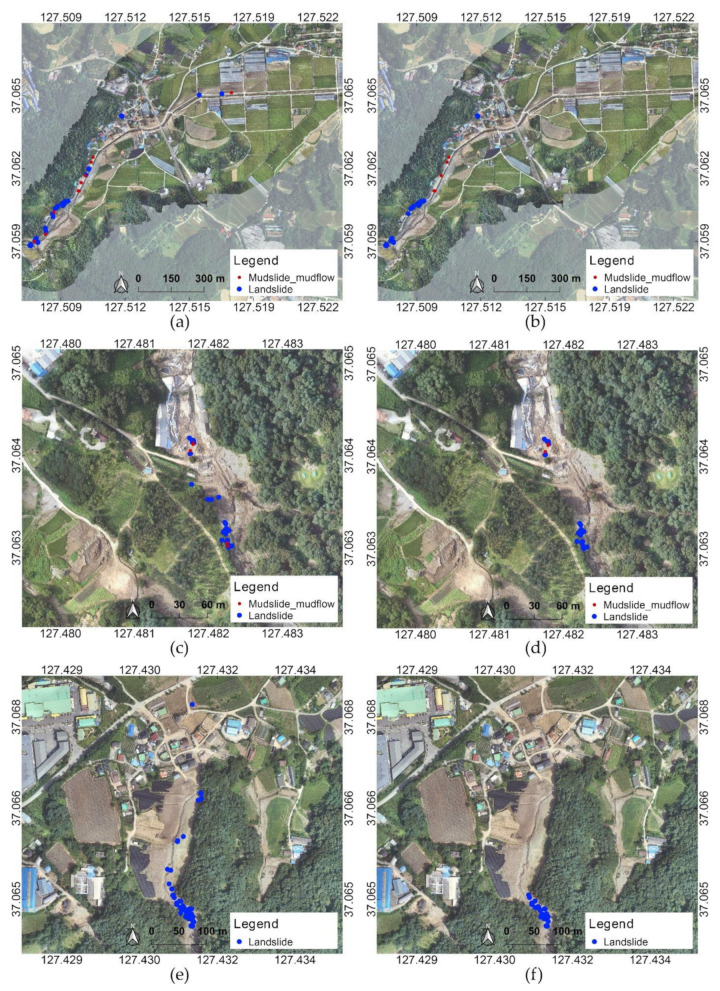
Distribution of results based on analysis of 24–25 August dataset: (**a**) results without hotspot analysis in Yul, (**b**) results with hotspot analysis in Yul, (**c**) results without hotspot analysis in Iljuk, (**d**) results with hotspot analysis in Iljuk, (**e**) results without hotspot analysis in Juksan, and (**f**) results with hotspot analysis in Juksan. The coordinates refer to WGS84 (EPSG: 4326).

The results obtained from the data collected on 24–25 August are shown in Figure 10. As with the results from the data collected on 5 August, when hotspot analysis was performed, it was confirmed that the main locations were separated according to the types of incident. For the detected incident types, landslide and mudslide mudflow, distribution patterns similar to the results for the 5 August dataset were obtained for the same regions. In the case of Juksan, the upper part of the area where the landslide occurred was identified as a severely damaged region both in the cases with and without hotspot analysis. However, the middle part of the damage was not detected in the result obtained with hotspot analysis, unlike in the result without hotspot analysis. The central region was not considered a hotspot because the slope was not steep, and the soil flowing down from the mountain was organized. In the images collected on 24–25 August, where restoration work was in progress, even the damaged site was classified as dirty contaminated and under construction. This is because there had been changes in the condition of the site, such as the drying of the soil, as images were collected nearly one month after the accident.

## 5. Discussion

In this study, a process for identifying major damage locations and principal images was proposed to support decision-making processes of disaster responders using image and sensor data acquired from smartphone applications. To verify the proposed process, an analysis was performed on data collected from Icheon-si and Anseong-si, which are in Gyeonggi-do, Korea, and which were damaged by heavy rainfall in 2020. Analysis was performed on the image and sensor data collected using the smartphone application developed in this study on 5 August, immediately after the accident, and during 24–25 August, when restoration activities were underway.

From the analysis results, when self-organizing map and graph-based clustering were used, incident types that better reflect on-site conditions were detected from the data collected on 5 August and during 24–25. In the case of data collected during 24–25 August, reliable filtering results were shown for most cases, even when filtering was performed based only on the confidence score. In addition, a more efficient filtering performance was observed after hotspot analysis. On 5 August, rainfall was ongoing at the study area, and the site was in a complicated state owing to collapse, flooding, and soil flow. For data collected in the field, filtering data only using the threshold of the confidence score are insufficient, and when the proposed methodology was applied, effective filtering was observed. A machine-learning-based approach can derive different results depending on the scene in an image. In addition, multiple damage types and aggravated weather conditions can complicate the scene and affect the analysis results. In general, disaster sites are not neat or simple, and the quality of crowdsourced data is unpredictable. Therefore, even if a crowd-sensing application is used in an emergency situation, it is necessary to filter the collected data to support decision-making.

The distributions of the data collected on 5 August and that collected on 24–25 August are different, but similar results were shown for major damaged areas. For data collected on 5 August and 24–25 August, there are differences in some types of incident such as collapsed, flooded, and earthquake because there are differences in the data collection environment and distribution of the collected data. However, for the critical incident types of landslide and mudslide mudflows, the damage locations were estimated in a similar manner. In addition, these results showed similar patterns when compared with the UAV orthoimages.

**Figure 11:**
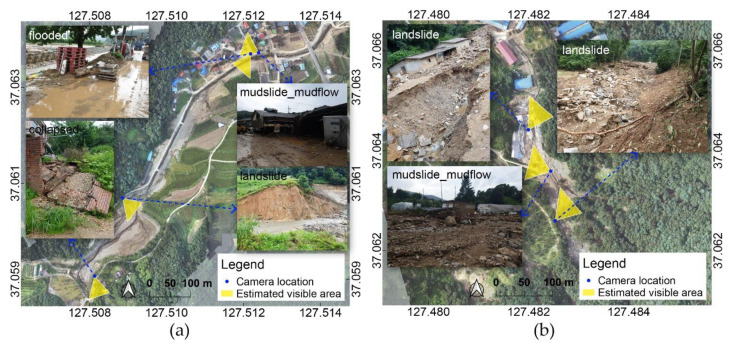
Example images from results analyzed with 5 August dataset: (**a**) Yul and (**b**) Iljuk. The coordinates refer to WGS84 (EPSG: 4326).

An example of the final images that were classified based on the collected data on 5 August is shown in Figure 11. Using the magnetometer and the gravity value collected from the mobile device, the azimuth value of the direction the camera is looking at can be estimated, with which the direction the camera is looking at on the map can be visualized. Examples show that images with significant damage were extracted from the classification.

This study aimed to deliver rapid situational information based on data collected during an emergency. It was verified that the proposed process can be used to effectively identify major damage locations from the classification of principal images from crowdsourced data in such situations. Smart devices are equipped with various sensors, such as gyroscopes, accelerometers, magnetometers, and GPS receivers. Accordingly, it is possible to improve the quantity and quality of data collected from the general public to support an emergency situation by using a crowd-sensing application. Therefore, in the future, research using various sensors to precisely analyze dynamic emergency situations should be conducted. Moreover, in crowd-sensing, the ability to grasp the user’s interest and motivation are treated as a major issue, and based on the proposed process, it is expected that users’ attention can be attracted by sharing analyzed collected information and participating in response activities, not just by reporting.

## 6. Conclusions

In recent years, the use of crowdsourced data has increased to enable the sharing of emergency situations and to support rapid response activities. However, research on extracting information through data processing and analysis based on data collected by focusing on emergency sites is insufficient. Therefore, we proposed a cloud computing-based data collection, processing, and analysis process that is based on crowd-sensing applications. To verify the suitability of the proposed process, an analysis was performed at three sites in Yul-myeon, Icheon-si and Iljuk-myeon and Juksan-myeon, Anseong-si, Gyeonggi-do, which were severely damaged by heavy rainfall in 2020. Based on the proposed methodology, of the numerous images collected, images reflecting serious damage situations could be effectively classified. In particular, for images collected immediately after a disaster, the existing deep learning-based classification was not stable, and the critical images were filtered through the proposed streamline. In addition, it was verified that locations of actual damage can be identified by making comparisons with UAV orthoimages. In the future, it is believed that it is necessary to apply images collected in actual accidents to better represent diverse types of incident, and it is expected that the proposed approach will support efficient decision-making in urgent situations.

## Figures and Tables

**Table 1:** Re-categorization of incident types.

Category [52,53]	Incident Type [16]	
	Main	Subsidiary
Infrastructural damage	Collapsed, Earthquake, Damaged	Blocked, Sinkhole
Damage to natural landscape	Landslide, Mudslide mudflow, Rockslide rockfall	Dirty contaminated, Under construction, Drought
Fire damage	Wildfire, On fire, Fire whirl, With smoke	Burned
Flood damage	Flooded	Tropical cyclone, Heavy rainfall, Tornado, Hailstorm, Storm surge, Derecho, Fog
Other damage	Snow-slide avalanche, Volcanic eruption, Nuclear explosion	Ice storm, Snow covered, Dust sandstorm, Thunderstorm, Dust devil, Traffic jam, Oil spill, Ship boat accident, Airplane accident, Car accident, Train accident, Bus accident, Bicycle accident, Motorcycle accident, Van accident, Truck accident

**Table 2:** Experiment dataset.

Study Area	$\mathrm{Census}\;\mathrm{Output}\;\mathrm{Area}\;({\mathrm{km}}^{2})$	Amount of Collected Data	
		5 August	24–25 August
Yul	7.5314	500	973
Iljuk	5.2513	434	204
Juksan	3.9827	-	567

**Table 3:** Result of incident detection through clustering approaches. Blue indicates the filtered results by Table 1.

Study Area (Date)	Cluster	K-Means Clustering	Agglomerative Clustering	Self-Organizing Map	Graph-Based Clustering
Yul (5 August)	1	Mudslide mudflow	Mudslide mudflow	Landslide	Landslide
	2	Landslide	Landslide	Mudslide mudflow	Mudslide mudflow
	3	Dirty contaminated	Dirty contaminated	Dirty contaminated	Dirty contaminated
	4	Collapsed	Collapsed	Collapsed	Collapsed
	5	Sinkhole	Drought	Flooded	Flooded
Iljuk (5 August)	1	Landslide	Landslide	Landslide	Landslide
	2	Mudslide mudflow	Mudslide mudflow	Mudslide mudflow	Mudslide mudflow
	3	Burned	Burned	Burned	Earthquake
	4	Earthquake	Earthquake	Earthquake	Burned
	5	Dirty contaminated	Dirty contaminated	Dirty contaminated	-
Yul (24 August)	1	Dirty contaminated	Dirty contaminated	Dirty contaminated	Dirty contaminated
	2	Under construction	Under construction	Under construction	Under construction
	3	Landslide	Landslide	Landslide	Landslide
	4	Drought	Drought	Drought	Drought
	5	Sinkhole	Mudslide mudflow	Mudslide mudflow	Mudslide mudflow
Iljuk (25 August)	1	Landslide	Landslide	Landslide	Landslide
	2	Mudslide mudflow	Mudslide mudflow	Mudslide mudflow	Mudslide mudflow
	3	Dirty contaminated	Dirty contaminated	Dirty contaminated	Burned
	4	Drought	Drought	Burned	Dirty contaminated
	5	Burned	-	Drought	Drought
Juksan (25 August)	1	Landslide	Landslide	Landslide	Landslide
	2	Under construction	Under construction	Under construction	Under construction
	3	Drought	Drought	Dirty contaminated	Drought
	4	Burned	Burned	Burned	Burned
	5	Dirty contaminated	-	-	Dirty contaminated

**Table 4:** Number of images classified by analysis (5 August dataset).

Incident	Threshold	Without Hotspot Analysis					With Hotspot Analysis				
		Very Heavy	Heavy	Moderate	Slight	None	Very Heavy	Heavy	Moderate	Slight	None
Yul- Landslide	0.1	175	75	11	15	1	74	46	1	3	1
	0.3	84	42	4	4	0	47	30	0	1	0
	0.5	46	20	2	0	0	31	17	0	0	0
	0.7	10	5	1	0	0	10	5	0	0	0
	0.9	0	0	0	0	0	0	0	0	0	0
Yul- Mudslide mudflow	0.1	218	44	5	8	0	110	20	0	1	0
	0.3	112	19	0	3	0	69	10	0	0	0
	0.5	56	13	0	1	0	39	9	0	0	0
	0.7	20	4	0	1	0	16	3	0	0	0
	0.9	2	0	0	0	0	2	0	0	0	0
Yul- Flooded	0.1	46	18	6	5	1	16	7	1	1	0
	0.3	7	7	0	0	0	3	6	0	0	0
	0.5	2	3	0	0	0	1	3	0	0	0
	0.7	0	0	0	0	0	0	0	0	0	0
	0.9	0	0	0	0	0	0	0	0	0	0
Yul- Collapsed	0.1	17	25	0	1	0	6	19	0	1	0
	0.3	9	8	0	0	0	4	8	0	0	0
	0.5	2	3	0	0	0	1	3	0	0	0
	0.7	2	0	0	0	0	1	0	0	0	0
	0.9	0	0	0	0	0	0	0	0	0	0
Iljuk- Landslide	0.1	300	34	6	0	0	59	0	2	0	0
	0.3	222	12	1	0	0	52	0	0	0	0
	0.5	129	7	1	0	0	39	0	0	0	0
	0.7	31	3	0	0	0	13	0	0	0	0
	0.9	0	0	0	0	0	0	0	0	0	0
Iljuk- Mudslide mudflow	0.1	242	19	3	1	0	83	6	0	1	0
	0.3	82	9	0	0	0	35	5	0	0	0
	0.5	27	5	0	0	0	10	2	0	0	0
	0.7	3	0	0	0	0	0	0	0	0	0
	0.9	0	0	0	0	0	0	0	0	0	0
Iljuk- Earthquake	0.1	64	5	0	1	0	0	0	0	0	0
	0.3	26	3	0	1	0	0	0	0	0	0
	0.5	12	0	0	0	0	0	0	0	0	0
	0.7	1	0	0	0	0	0	0	0	0	0
	0.9	0	0	0	0	0	0	0	0	0	0

**Table 5:** Number of images classified by analysis (24–25 August dataset).

Incident	Threshold	Without Hotspot Analysis					With Hotspot Analysis				
		Very Heavy	Heavy	Moderate	Slight	None	Very Heavy	Heavy	Moderate	Slight	None
Yul- Landslide	0.1	93	90	59	24	4	49	41	11	11	2
	0.3	40	38	22	5	0	30	24	5	2	0
	0.5	15	19	6	0	0	15	15	1	0	0
	0.7	1	5	0	0	0	1	5	0	0	0
	0.9	0	0	0	0	0	0	0	0	0	0
Yul- Mudslide mudflow	0.1	56	37	51	7	0	13	7	20	1	0
	0.3	11	12	15	1	0	4	3	12	1	0
	0.5	3	4	4	1	0	1	1	4	1	0
	0.7	1	2	1	0	0	0	1	1	0	0
	0.9	0	0	0	0	0	0	0	0	0	0
Iljuk- Landslide	0.1	50	70	4	1	3	44	49	1	0	0
	0.3	38	44	2	0	0	33	34	1	0	0
	0.5	15	20	0	0	0	12	17	0	0	0
	0.7	3	1	0	0	0	2	0	0	0	0
	0.9	0	0	0	0	0	0	0	0	0	0
Iljuk- Mudslide mudflow	0.1	23	18	3	0	1	20	6	1	0	0
	0.3	8	8	1	0	0	7	4	0	0	0
	0.5	3	2	0	0	0	3	0	0	0	0
	0.7	0	0	0	0	0	0	0	0	0	0
	0.9	0	0	0	0	0	0	0	0	0	0
Juksan- Landslide	0.1	261	54	18	4	2	50	0	0	0	0
	0.3	170	25	6	1	1	50	0	0	0	0
	0.5	106	5	1	0	0	50	0	0	0	0
	0.7	64	0	0	0	0	50	0	0	0	0
	0.9	0	0	0	0	0	0	0	0	0	0
